# Adult Sarcomas with NTRK Fusions: Clinicopathologic and Genomic Heterogeneity

**DOI:** 10.3390/cancers18152528

**Published:** 2026-08-06

**Authors:** Michael Schwartz, Kieran Sweeney, Steven C. Smith, Kartik Angara, Celia Reynolds, Alberto S. Pappo, Andrew Elliott, Matthew J. Oberley, Mark G. Evans, Armita Bahrami

**Affiliations:** 1 Department of Pathology and Laboratory Medicine, Emory University School of Medicine, Atlanta, GA 30322, USA; michael.schwartz@emory.edu; 2 Winship Cancer Institute of Emory University, Atlanta, GA 30322, USA; 3 Caris Life Sciences, Phoenix, AZ 85040, USAkangara@carisls.com (K.A.); moberley@carisls.com (M.J.O.); mevans@carisls.com (M.G.E.); 4 Department of Pathology, University of Virginia School of Medicine, Charlottesville, VA 22908, USA; scs8u@uvahealth.org; 5 Department of Radiation Oncology, Atrium Health Wake Forest Baptist, Winston-Salem, NC 27157, USA; celia.reynolds@advocatehealth.org; 6 Department of Oncology, St. Jude Children’s Research Hospital, Memphis, TN 38105, USA

**Keywords:** NTRK-rearranged sarcomas, TRK inhibitors, NTRK gene fusions, sarcoma genomics, next-generation sequencing, precision oncology, fusion-driven tumors

## Abstract

Background: NTRK gene fusions are established oncogenic drivers in a diverse spectrum of mesenchymal neoplasms. Although classically described in pediatric entities, NTRK-rearranged sarcomas also occur in adults, where their clinicopathologic features, genomic context, and response to TRK inhibition are less well characterized. Methods: We retrospectively queried a national referral genomics database to identify sarcomas in patients >18 years harboring pathogenic *NTRK1*, *NTRK2*, or *NTRK3* fusions. Gastrointestinal stromal tumors, duplicate specimens, and cases lacking digitized hematoxylin and eosin slides were excluded. Fusions were identified by whole-transcriptome sequencing, co-occurring genomic alterations by exome-based sequencing, and real-world survival and time on TRK inhibitor therapy were derived from linked insurance claims data; fusion-negative sarcomas and NTRK-rearranged non-sarcoma tumors from the same database served as comparison cohorts. Results: Among 13,040 profiled sarcomas, 19 adult tumors with pathogenic NTRK fusions were identified (median age, 43 years; range, 21–77), most of which were high grade (68%) and advanced stage (63% stage IV). Histology was heterogeneous, including spindle cell sarcoma (53%), pleomorphic sarcoma (21%), and tumors corresponding to defined entities such as *NF1*-associated malignant peripheral nerve sheath tumor and *MDM2*-amplified dedifferentiated liposarcoma (11% each). *NTRK1* and *NTRK3* fusions were equally frequent (9 cases each); fusion partners were diverse, with *TPM3* (*n* = 5), *EML4* (*n* = 2), and *TFG* (*n* = 2) recurrent and other partners non-recurrent. Additional genomic alterations were common and heterogeneous (72%), including high genome-wide loss of heterozygosity and infrequent but recurrent alterations involving the *TERT* promoter, *NF1*, and *RB1*. All evaluable tumors showed transcriptional activation of the NTRK fusion and increased MAPK pathway activity compared with fusion-negative sarcomas. Nine patients received TRK inhibitors; median time on larotrectinib was 12.5 months, similar to that observed in NTRK-rearranged non-sarcoma tumors, but treatment duration was variable. Conclusions: Adult sarcomas harboring NTRK fusions are rare, morphologically heterogeneous, and biologically diverse. NTRK fusion status alone may not fully capture oncogenic dependence and should be interpreted within the broader clinicopathologic and genomic context.

## Simple Summary

Adult sarcomas harboring NTRK fusions are rare and represent a biologically heterogeneous group with diverse morphologic, genomic, and clinical features. In this retrospective cohort of 19 adult sarcomas with NTRK fusions, we found substantial variation in fusion partners, secondary genomic alterations, and histologic appearance, including tumors arising within established entities such as *NF1*-associated malignant peripheral nerve sheath tumor and *MDM2*-amplified dedifferentiated liposarcoma. Although all evaluable tumors showed transcriptional activation of the fusion and increased MAPK pathway activity, clinical benefit from TRK inhibition was variable. These findings suggest that NTRK fusions may function in different biological contexts across adult sarcomas and that fusion status alone may not fully capture oncogenic dependence or therapeutic susceptibility to TRK inhibitors. Thus, adult sarcomas harboring NTRK fusions should be interpreted in the broader clinicopathologic and genomic context.

## 1. Background

Gene fusions involving the neurotrophic tyrosine receptor kinase genes (*NTRK1*, *NTRK2*, and *NTRK3*) are established oncogenic drivers across a wide range of tumor types. Recurrent *NTRK3* fusions were first recognized in well-defined pediatric entities, such as infantile fibrosarcoma, congenital mesoblastic nephroma, and secretory carcinoma of the breast and salivary glands [1,2,3,4]. In these tumors, NTRK fusions represent the primary oncogenic driver and are associated with characteristic clinicopathologic features.

More recently, NTRK rearrangements have been identified in a broader spectrum of mesenchymal neoplasms beyond infantile fibrosarcoma [5,6,7,8,9]. These NTRK-rearranged mesenchymal tumors are best recognized in children and young adults and are generally regarded as biologically intermediate neoplasms rather than frankly high-grade sarcomas, often pursuing a course that, while capable of local recurrence and occasional local aggression, generally lacks the metastatic potential typical of conventional high-grade sarcomas [5,10,11]. In contrast, high-grade sarcomas harboring NTRK fusions remain less well defined and appear to be uncommon [5,7,11]. In adults particularly, NTRK-rearranged mesenchymal tumors remain incompletely characterized, with reported cases showing wide variation in anatomic site, morphology, and clinical behavior, ranging from indolent lesions to aggressive sarcomas with metastatic potential [12,13,14,15,16], raising an important biological question: whether NTRK fusions always function as primary oncogenic drivers or may instead occur as cooperative events within a more complex genomic background.

The identification of NTRK rearrangements has significant therapeutic implications, as selective TRK inhibitors (larotrectinib and entrectinib) have demonstrated high response rates in tumor-agnostic studies [17,18,19]. Clinical responses have also been documented in numerous case reports and case series [20]. However, data specific to sarcomas remain limited and are derived largely from subset analyses within broader TRK inhibitor studies [21].

Using a large, clinically annotated dataset from a national referral genomic laboratory, we performed a clinicopathologic and genomic analysis of adult sarcomas harboring pathogenic NTRK fusions. This study included adult sarcomas irrespective of histologic subtype, from tumors lacking defining molecular alterations to recognized sarcoma entities in which an NTRK fusion may coexist with the canonical genomic driver. This design allowed us to investigate whether the oncogenic significance of NTRK fusions in adult sarcomas is influenced by the broader genomic context. The objectives of this study were to: (1) define the clinicopathologic spectrum of adult sarcomas harboring NTRK fusions across diverse histologic subtypes; (2) characterize co-occurring genomic alterations and the broader oncogenic context; and (3) evaluate clinical outcomes following TRK inhibitor therapy and explore whether NTRK fusion status alone predicts oncogenic dependence.

## 2. Methods

The study was conducted in accordance with the Declaration of Helsinki, the Belmont Report, and the U.S. Common Rule. In keeping with 45 CFR 46.104(d)(4), the study utilized retrospective, deidentified clinical data and was deemed exempt from IRB review with a waiver of informed consent. Exempt status was determined by the Western Copernicus Group Institutional Review Board (WCG IRB).

The genomic database at Caris Life Sciences was retrospectively queried to identify sarcoma specimens harboring a pathogenic *NTRK1*, *NTRK2*, or *NTRK3* gene fusion. A total of 13,040 sequenced sarcoma cases were screened. Only adult cases were included; pediatric patients (<18 years), infantile fibrosarcoma, and gastrointestinal stromal tumor (GIST) were excluded. Cases lacking hematoxylin and eosin (H&E) slides for histopathologic review were also excluded. For patients with multiple specimens, only the earliest chronologically analyzed sample was retained. Cases were retained regardless of histologic classification, including recognized sarcoma entities such as malignant peripheral nerve sheath tumor (MPNST) and dedifferentiated liposarcoma. This approach reflects the tumor-agnostic paradigm of NTRK-targeted therapy, in which any tumor harboring a pathogenic NTRK fusion may be eligible for TRK inhibitor treatment regardless of its primary histologic diagnosis. Following genomic case identification and study cohort selection, all eligible cases underwent retrospective clinical and histopathologic review.

### 2.1. Molecular Profiling

H&E-stained, formalin-fixed, paraffin-embedded (FFPE) slides of the patients’ tumors were reviewed by a board-certified pathologist or a trained pathologist assistant to confirm tumor content and guide manual microdissection for targeted tissue enrichment.

#### 2.1.1. DNA and RNA Sequencing

Genomic profiling was performed using clinically validated next-generation sequencing assays. Because cases were accrued over several years, sequencing platforms and analytical pipelines evolved over time. DNA profiling in earlier phases was performed using a targeted 592-gene panel and subsequently transitioned to whole exome sequencing (WES) with targeted enrichment of clinically relevant genes. Whole transcriptome sequencing (WTS) was performed in parallel during earlier phases of testing. In later cases, DNA and RNA sequencing were performed concurrently from a single nucleic acid extraction using a hybrid DNA/RNA assay. Detailed descriptions of nucleic acid extraction, sequencing platforms, bioinformatic pipelines, and variant interpretation criteria are provided in Supplementary Material [22,23].

#### 2.1.2. Fusion Detection

Gene fusions and splice variants were identified from RNA sequencing data using clinically validated fusion-calling algorithms with predefined read-support thresholds. Fusion calls were classified as detected or not detected based on established criteria for junction and spanning reads. Gene expression levels were quantified as transcripts per million (TPM). Full methodological details, including detection thresholds and classification rules, are provided in Supplementary Material.

#### 2.1.3. Copy Number Amplifications

Copy number alterations were assessed using validated computational methods applied to sequencing data. Amplification status was determined for a predefined set of clinically relevant genes using assay-validated, gene-specific thresholds. A detailed description of copy number calling methods and thresholds is provided in Supplementary Material.

#### 2.1.4. Loss of Heterozygosity

Genome-wide loss of heterozygosity (LOH) was evaluated using single nucleotide polymorphism-based segmentation of autosomal chromosomes and comparison with reference control data. LOH status was categorized using predefined percentage thresholds, with indeterminate calls assigned in cases of insufficient coverage or depth. Full details of LOH assessment, segmentation strategy, statistical testing, and classification criteria are provided in Supplementary Material.

#### 2.1.5. Tumor Mutational Burden

Tumor mutational burden (TMB) was calculated as the number of qualifying somatic mutations per megabase of coding sequence analyzed, using assay-specific genomic footprints. Variant filtering and classification followed established clinical standards. A cutoff of ≥10 mutations per megabase was applied to define high TMB, consistent with published clinical trial data [24]. Samples failing to meet minimum coverage requirements were classified as indeterminate. Detailed TMB methodology is provided in Supplementary Material. Caris Life Sciences is a participant in the Friends of Cancer Research TMB Harmonization Project [25].

### 2.2. Clinical Assessment

Clinical data were extracted from accompanying pathology reports and available clinical records. Variables collected included patient age and sex, primary tumor site, tumor size, specimen type (primary tumor, local recurrent, or metastatic lesion), stage, referring diagnosis, treatment modalities, and clinical outcome.

### 2.3. Pathologic Review and Assessment

Histopathologic evaluation of digitized H&E-stained slides included assessment of tumor architecture, cytologic features, mitotic activity, necrosis, and histologic grade. Tumors were classified according to their predominant morphology as spindle cell sarcoma, NOS, or pleomorphic sarcoma, with additional descriptors (e.g., myxoid features) recorded where applicable. Where available, immunophenotypic and molecular findings were integrated into the final pathologic classification. Histologic grade was assigned according to the FNCLCC grading system and reported as low (grade 1), intermediate (grade 2), or high (grade 3).

### 2.4. Survival Analysis

Real-world overall survival data were derived from deidentified insurance claims databases. Overall survival was calculated from the date of tissue acquisition, which served as the index date for molecular profiling, to death or last clinical contact, with insurance claims used to determine survival status. In the present analysis, a 365-day threshold was applied as a conservative criterion to minimize false presumption of death, consistent with a previously published methodological framework [26]. Patients with no insurance claims or documented clinical contact for at least 365 days before the most recent data update were presumed deceased; those without confirmed death but with documented clinical activity within the same window were censored. Time on treatment was calculated for patients receiving TRK inhibitor therapy. Two external comparator cohorts were derived from the same national real-world genomic database: (1) patients with NTRK-rearranged non-sarcoma solid tumors, to contextualize larotrectinib treatment duration relative to the sarcoma cohort; and (2) patients with sarcomas without a pathogenic NTRK fusion who received larotrectinib, serving as a fusion-negative reference. Survival estimates were generated using Kaplan–Meier methodology, with hazard ratios calculated using Cox proportional hazards models and statistical significance assessed by log-rank testing.

## 3. Results

### 3.1. Clinical and Demographic Characteristics

Among 13,040 sequenced sarcomas, 43 (0.33%) harbored a pathogenic *NTRK1*, *NTRK2*, or *NTRK3* fusion. After excluding pediatric cases, tumors classified as infantile fibrosarcoma or GIST, duplicate samples, and cases lacking digitized H&E stained slides for pathologic review, a final cohort of 19 unique adult NTRK-rearranged sarcomas was identified.

The median patient age was 43 years (range, 21–77 years; Table 1). Twelve patients (63%) were female and seven (37%) were male. Race was self-reported as White in 7 patients (36.8%), Black or African American in 3 (15.8%), and unknown or other in 9 (47.4%).

**Figure 1:**
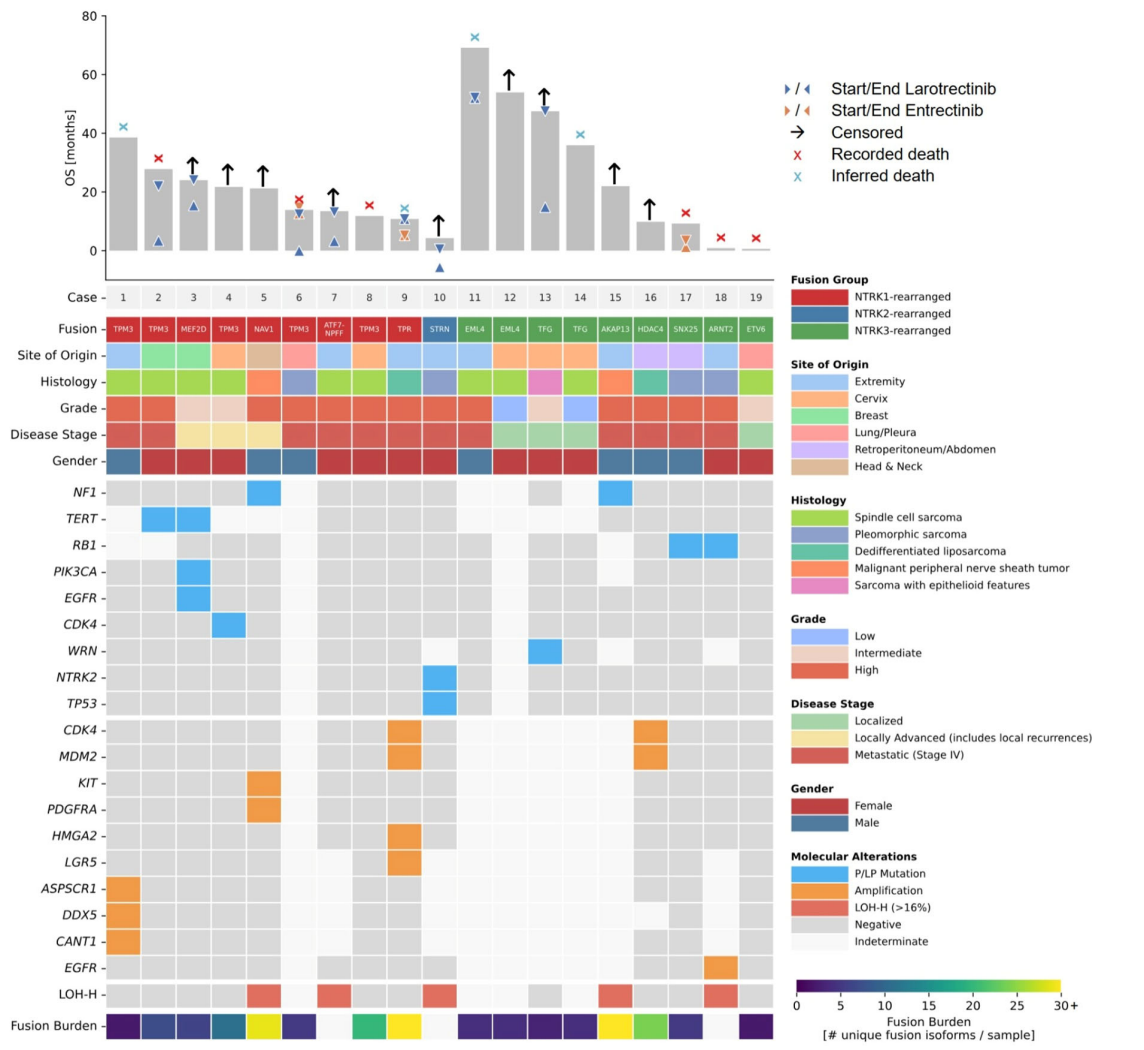
Genomic alterations and clinical course of adult sarcomas with NTRK fusions. Swimmer plot (top) and oncoprint (bottom) summarizing clinical course, selected clinicopathologic features, and genomic alterations in 19 adult sarcomas harboring NTRK fusions. In the oncoprint, each column represents an individual tumor and rows denote NTRK fusion partners and selected genomic alterations. Pathogenic or likely pathogenic mutations are shown in rows toward the top and copy-number amplifications in rows toward the bottom, so some genes appear in more than one row. Cells in the fusion row indicate specific NTRK fusion partners. Fusion burden is defined as the number of unique fusion isoforms detected per tumor. Colors denote alteration classes as indicated in the key. Clinical annotations, including histologic subtype and tumor grade, are shown above the oncoprint. Abbreviations: LOH, loss of heterozygosity; P/LP, pathogenic/likely pathogenic; #, number.

Primary tumors arose across diverse anatomic sites (Figure 1). Soft tissue primaries accounted for 8 of 19 cases (42%), including 7 tumors in the extremities and 1 in the head and neck region. Gynecologic primaries were also common, with 5 of 19 tumors (26%) originating in the cervix. Two cases (11%) arose in the breast, two (11%) in the lung or pleura, and two (11%) in the retroperitoneum or abdomen. Tumor size ranged from 3.8 to 46 cm, with a median size of 7.9 cm.

Specimens were obtained from primary tumors (8/19, 42%), metastatic sites (8/19, 42%), and local recurrences (3/19, 16%). Most patients had advanced disease: 12 of 19 (63%) had stage IV disease, 3 (16%) locally advanced or recurrent disease, and 4 (21%) localized disease (Figure 1). The cohort was also analyzed according to fusion subtype, with cases grouped as *NTRK1*- or *NTRK3*-rearranged (Table 1); the single *NTRK2*-rearranged case was excluded from these subgroup analyses. No statistically significant differences were detected between subgroups with respect to age, sex, primary tumor site, histologic category, or histologic grade. Within this cohort, cases in the *NTRK1*-rearranged subgroup more frequently had locally advanced, recurrent, or metastatic disease than those in the *NTRK3*-rearranged subgroup (Fisher exact test, localized vs. nonlocalized stage, *p* = 0.022).

### 3.2. Pathologic Findings

The original referring diagnoses encompassed a broad spectrum of predominantly high-grade sarcomas with considerable diagnostic heterogeneity. Following centralized histopathologic review, tumors were classified according to their predominant morphology. Spindle cell sarcoma was the predominant pattern (10/19, 53%), followed by pleomorphic sarcoma (4/19, 21%), with one tumor classified separately as a sarcoma with predominant epithelioid morphology (Tables S1 and S2). Four tumors fulfilled diagnostic criteria for recognized sarcoma entities, including two MPNSTs and two dedifferentiated liposarcomas (11% each). Both MPNSTs occurred in patients with neurofibromatosis type 1 (NF1) and were associated with neurofibromas, whereas both dedifferentiated liposarcomas demonstrated *MDM2* amplification with an identifiable well-differentiated liposarcoma component.

**Figure 2:**
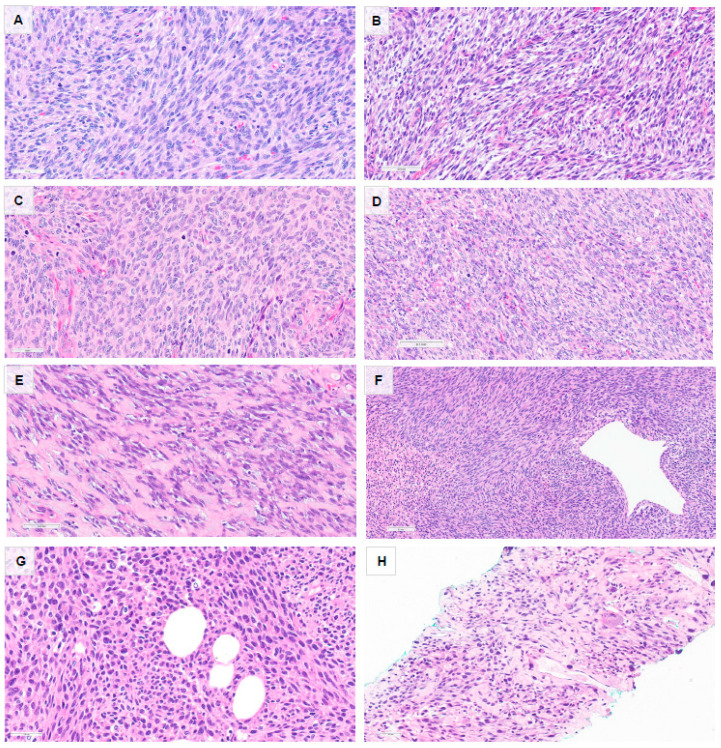
Representative histologic features of adult sarcomas with *NTRK1* fusions. (**A**–**E**) High- and intermediate-grade spindle cell sarcomas with *NTRK1* rearrangements, showing a predominant pattern of fascicular growth of relatively monomorphic spindle cells ((**A**–**D**): high grade; (**E**): intermediate grade). (**F**) *NF1*-associated malignant peripheral nerve sheath tumor with an *NTRK1* fusion. (**G**) *MDM2*-amplified dedifferentiated liposarcoma with an *NTRK1* fusion. (**H**) *NTRK1*-rearranged high-grade undifferentiated pleomorphic sarcoma.

**Figure 3:**
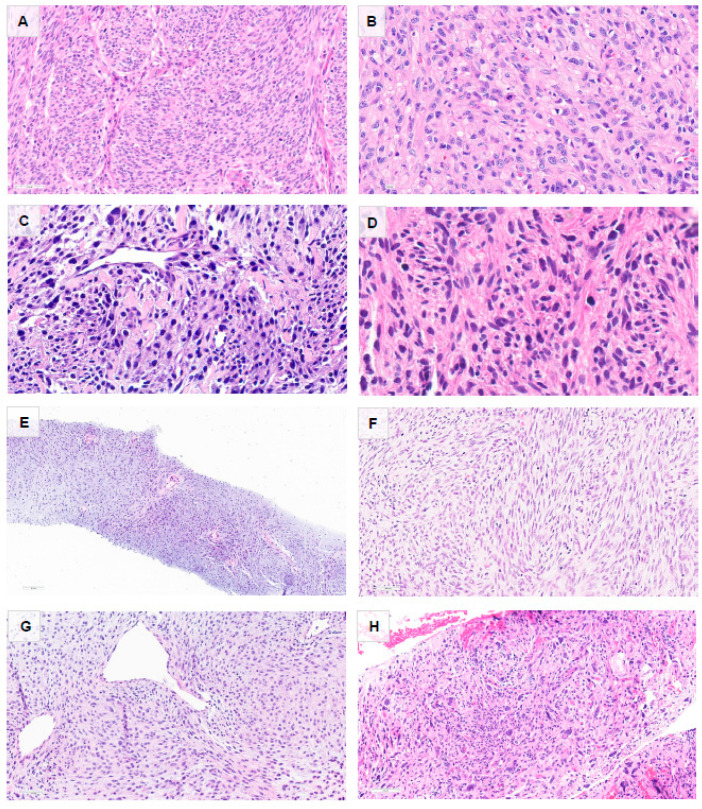
Representative histologic features of adult sarcomas with *NTRK3* fusions showing the morphologic heterogeneity of *NTRK3*-rearranged sarcomas. (**A**) *NTRK3*-rearranged high-grade spindle cell sarcoma. (**B**) *NTRK3*-rearranged intermediate-grade sarcoma with epithelioid features. (**C**,**D**) *NTRK3*-rearranged high-grade undifferentiated pleomorphic sarcomas. (**E**) *NTRK3*-rearranged intermediate-grade spindle cell sarcoma with myxoid features. (**F**) *NTRK3*-rearranged low-grade spindle cell sarcoma. (**G**) *NF1*-associated malignant peripheral nerve sheath tumor with an *NTRK3* fusion. (**H**) *MDM2*-amplified dedifferentiated liposarcoma with an *NTRK3* fusion.

Tumor morphology did not show clear segregation by fusion type (Table 1). However, *NTRK1*-rearranged tumors were mostly classified as spindle cell sarcoma, whereas *NTRK3*-rearranged tumors were more morphologically heterogeneous, encompassing spindle cell sarcoma, pleomorphic sarcoma, and one case showing epithelioid morphology (Figure 2 and Figure 3). Most tumors were high grade; using the FNCLCC system, 13/19 (68%) were grade 3, 4/19 (21%) grade 2, and 2/19 (11%) grade 1.

### 3.3. Molecular Findings

#### 3.3.1. Fusion Gene Distribution

Fusions involving *NTRK1* and *NTRK3* occurred with equal frequency in the cohort, with 9 cases each, whereas only a single tumor harbored an *NTRK2* fusion (Table 2). Among *NTRK1*-rearranged tumors, *TPM3* was the most frequent fusion partner, identified in 5 of 9 cases; the remaining partners (*ATF7*, *NPFF*, *MEF2D*, *TPR*, and *NAV1*) each occurred once. Most *TPM3:NTRK1* tumors (4 of 5) were classified as spindle cell sarcoma, NOS, with only one showing pleomorphic morphology. *NTRK3*-rearranged tumors showed greater diversity of fusion partners, with seven distinct partners identified across nine tumors. Recurrent partners included *TFG* and *EML4* (two cases each), whereas *AKAP13*, *ARNT2*, *ETV6*, *HDAC4*, and *SNX25* each occurred once.

*NTRK1* fusions were predicted to originate mostly from intrachromosomal inversions, whereas *NTRK3* fusions were predicted to result more frequently from interchromosomal translocations (7 of 9; Fisher exact test, *p* < 0.05). Overall, there was no overlap in fusion partners between *NTRK1*- and *NTRK3*-rearranged tumors.

#### 3.3.2. Secondary Molecular Alterations

Secondary genomic alterations were heterogeneous and lacked a dominant recurrent pattern (Figure 1). High-level genomic loss of heterozygosity (LOH-high; >16%, assay-defined threshold) was identified in 5 of 15 evaluable tumors. Two of these cases were MPNSTs arising in patients with neurofibromatosis type 1, while two occurred in pleomorphic sarcomas.

Recurrent alterations were uncommon. Mutations involving the *TERT* promoter, *NF1*, and *RB1* were each identified in two cases, whereas other pathogenic or likely pathogenic variants occurred in isolated tumors (Figure 1). In this small cohort, *RB1* mutations occurred only in *NTRK3*-rearranged tumors, whereas *TERT* promoter mutations occurred only in *NTRK1*-rearranged tumors, both arising in the breast. Two tumors showed co-amplification of *MDM2* and *CDK4*, both in dedifferentiated liposarcomas, as expected for this tumor type. TMB was low across the cohort (median, 3 mutations per megabase), with all cases below 10 mutations per megabase (Figure S1A). No statistically significant differences in secondary genomic alterations were observed between *NTRK1*- and *NTRK3*-rearranged subgroups.

Fusion burden, defined as the number of distinct fusion events or isoforms per sample, varied across tumors. A subset of cases (5 of 17 evaluable tumors) showed a higher fusion burden, indicative of a higher background of structural rearrangements (Figure 1). These tumors included *NF1*-associated MPNST and *MDM2*-amplified dedifferentiated liposarcoma. No consistent correlation was observed between fusion burden and NTRK fusion type. The single *NTRK2*-rearranged tumor also harbored a kinase-domain *NTRK2* mutation (G639R) that was detected post TRK-inhibitor therapy, indicative of a resistance mutation.

#### 3.3.3. Gene Expression Analysis

**Figure 4:**
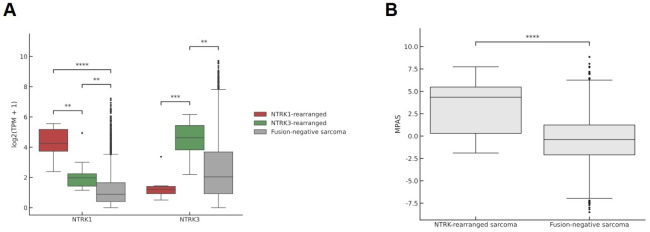
Fusion-specific NTRK expression and MAPK pathway activation in NTRK-rearranged sarcomas. (**A**) RNA expression of *NTRK1* and *NTRK3*, measured as transcripts per million (TPM), in *NTRK1*-rearranged and *NTRK3*-rearranged sarcomas (*n* = 8 each) compared with NTRK fusion-negative sarcomas (*n* = 8940). (**B**) MAPK pathway activity score (MPAS) in NTRK-rearranged sarcomas (*n* = 16) compared with fusion-negative sarcomas (*n* = 8940). Solid circles in boxplots represent outliers. Stars indicate statistical significance as follows: **** *p* < 0.0001; *** *p* < 0.001; ** *p* < 0.01.

Gene expression data were available for 16 of 19 tumors. Expression of *NTRK1* and *NTRK3* was concordant with fusion status. *NTRK1*-rearranged tumors showed higher *NTRK1* expression compared with *NTRK3*-rearranged tumors (median 18.1 vs. 2.9 TPM; Mann–Whitney U test, *p* = 0.0047) and fusion-negative sarcomas (median 0.9 TPM; *p* < 0.0001; Figure 4A). Conversely, *NTRK3*-rearranged tumors showed higher *NTRK3* expression than *NTRK1*-rearranged tumors (median 25.5 vs. 1.3 TPM; *p* = 0.0006) and fusion-negative sarcomas (median 3.1 TPM; *p* = 0.0023).

MAPK pathway activity scores were higher in NTRK1/3 fusion-positive tumors compared with fusion-negative sarcomas (median 4.35 vs. −0.39; *p* < 0.0001; Figure 4B). No statistically significant difference in MAPK pathway activity scores was observed between *NTRK1*- and *NTRK3*- Fusion-positive tumors (Figure S1B).

#### 3.3.4. Outcomes and Response to TRK Inhibition

Real-world overall survival, calculated from the date of tissue collection (index date) to death or last clinical contact, varied across the cohort (Figure 1). Six patients had a recorded date of death. Among the remaining patients without a documented death, 9 were censored at their last clinical activity within 365 days of the most recent data update.

When stratified by stage, patients with metastatic disease had shorter real-world overall survival than those with localized or locally advanced disease (median 14.8 vs. 35.9 months), although the difference was not statistically significant (log-rank *p* = 0.43; HR 1.65, 95% CI 0.48–5.70). Similarly, when stratified by histologic grade, intermediate- and high-grade tumors had shorter real-world overall survival compared with low-grade tumors (median 21.2 vs. 53.9 months), but this difference was not statistically significant (log-rank *p* = 0.27; HR 2.09, 95% CI 0.55–8.03).

Nine of 19 patients were treated with a TRK inhibitor, including six who received larotrectinib monotherapy, one entrectinib monotherapy, and two who received sequential treatment with both agents (Figure 1). Time on treatment was variable (median 12.5 months). Several patients had prolonged treatment durations, suggesting possible durable clinical benefit, whereas others had modest or short treatment courses, including a patient with *MDM2/CDK4* amplification who had only brief exposure to both larotrectinib and entrectinib. Across treated cases, shorter treatment durations were often observed in tumors with co-occurring alterations such as *TP53* and *RB1*, although the small number of treated patients precluded definitive assessment of any association between genomic context and TRK inhibitor benefit.

**Figure 5:**
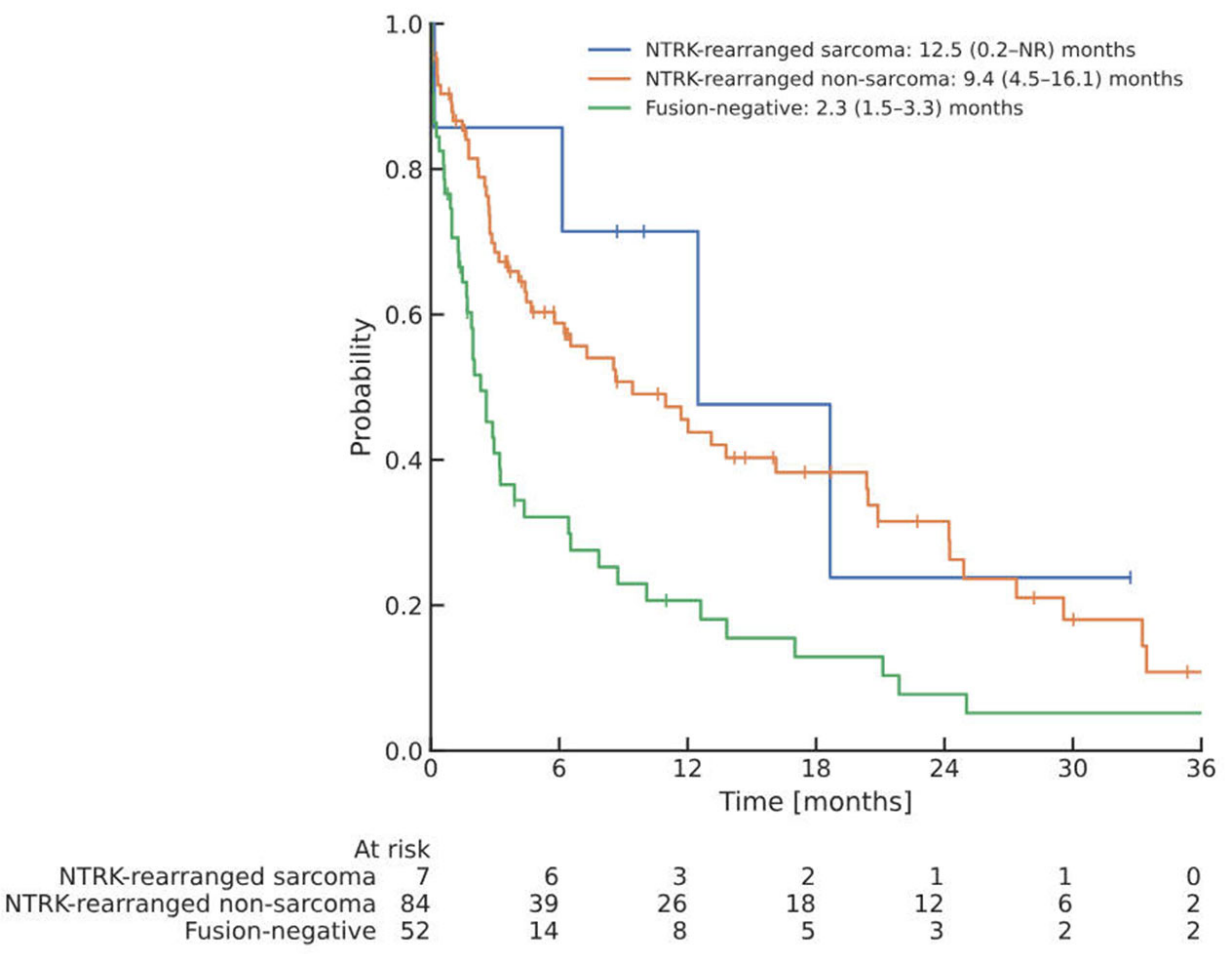
Time on larotrectinib treatment by NTRK fusion status and tumor category. Kaplan–Meier curves depicting time on larotrectinib treatment in three cohorts: patients with NTRK-rearranged sarcomas (blue), patients with NTRK-rearranged non-sarcoma tumors (orange), and patients without pathogenic NTRK fusions (fusion-negative; green). Median time on larotrectinib (with 95% confidence intervals) is shown in the legend for each cohort. The numbers shown below the *x*-axis indicate, for each time point, how many patients in each cohort remained at risk (i.e., still receiving larotrectinib and not yet censored or off treatment) at that time.

Median time on larotrectinib for patients with NTRK-rearranged sarcomas was similar to that observed in patients with NTRK-rearranged non-sarcoma solid tumors (12.5 vs. 9.4 months; *p* = 0.56) and longer than in sarcoma patients without a pathogenic NTRK fusion who received larotrectinib (2.3 months; *p* = 0.054) (Figure 5). Together, these findings suggest that larotrectinib can provide meaningful benefit in a subset of NTRK-rearranged sarcomas, although treatment duration is variable and robust predictors of benefit could not be defined in this small series.

## 4. Discussion

Our analysis demonstrates that adult sarcomas harboring NTRK fusions represent a biologically heterogeneous group spanning diverse morphologic, genomic, and clinical contexts. Collectively, our findings suggest that the biological significance of these fusions cannot be inferred from fusion status alone but is influenced by the accompanying clinicopathologic and genomic landscape.

This study confirms that NTRK fusion-positive sarcomas are rare in adults. These tumors showed no consistent morphologic features that reliably distinguished them from other sarcomas and exhibited a broad spectrum of histologic appearances, frequently with high-grade features. These observations are consistent with previous reports [7,15,27] and support the use of broad-panel next-generation sequencing in the evaluation of histologically ambiguous adult sarcomas, where reliance on morphology alone may delay recognition of actionable alterations. Despite this overall morphologic heterogeneity, subtle patterns emerged within molecularly defined subgroups. Within the *NTRK1* subgroup, *TPM3::NTRK1* was the predominant fusion (5 of 9 tumors), with four of these tumors showing a spindle cell sarcoma phenotype. Although based on small numbers, this association raises the possibility that recurrent *TPM3::NTRK1* fusions contribute to the relatively uniform spindle cell morphology observed in a subset of adult *NTRK1*-rearranged sarcomas, consistent with reports of fibrosarcoma-like spindle cell morphology in *NTRK1*-rearranged gynecologic sarcomas [14,16].

*NTRK1* and *NTRK3* fusions occurred with similar frequency in this cohort, whereas *NTRK2* rearrangements were uncommon, consistent with previous adult series [12,21]. *NTRK1* fusions more commonly resulted from intrachromosomal inversions, whereas *NTRK3* fusions arose through interchromosomal translocations, in keeping with previously described structural patterns of *NTRK1* and *NTRK3* rearrangements [28]. Fusion partners were diverse and included both well-recognized (e.g., *TPM3::NTRK1*, *TPR::NTRK1*, and *EML4::NTRK3*) and less commonly reported partners [15,16,29]. Several fusion partners identified in this cohort have only rarely been reported in sarcomas, whereas others, including *ARNT2* and *SNX25*, have not previously been described, further expanding the molecular spectrum of adult NTRK-rearranged sarcomas. *ETV6*-associated fusions, highly characteristic of pediatric tumors such as infantile fibrosarcoma and secretory carcinoma, were distinctly rare, underscoring the biologic differences between pediatric and adult NTRK-rearranged tumors [5,7].

The extent to which NTRK fusion type independently influences clinical behavior remains unclear. The predominance of high-grade, advanced-stage tumors likely reflects referral bias in a cohort enriched for clinically aggressive or diagnostically challenging sarcomas submitted for comprehensive molecular profiling. Consequently, this cohort is not directly comparable to the predominantly low-grade NTRK-rearranged spindle cell neoplasms reported in the literature. Although *NTRK1*-rearranged tumors more frequently presented with nonlocalized disease than *NTRK3*-rearranged tumors, this observation should not be interpreted as evidence of an intrinsic biological difference between fusion types.

Beyond the NTRK fusion, secondary genomic alterations were heterogeneous across the cohort. High-level LOH and alterations affecting cell-cycle regulation and telomere/senescence pathways (*RB1* and *TERT*) were identified in a subset of tumors. Previous studies have likewise demonstrated that adult NTRK-rearranged sarcomas may harbor complex genomic landscapes, including widespread copy number alterations and mutations involving genes such as *CDKN2A*, *NF2*, *TP53*, and *MYC*, which may influence tumor biology and therapeutic response [12,13,30]. Collectively, these observations suggest that secondary genomic alterations may contribute to tumor progression and the aggressive clinicopathologic features observed in a subset of adult NTRK-rearranged sarcomas. In tumors treated with TRK inhibitors, secondary genomic alterations and activation of downstream pathways have also been implicated in acquired resistance to TRK inhibition [19,31,32].

Importantly, a subset of tumors in this cohort arose within known sarcoma entities, including *NF1*-associated MPNST and *MDM2*-amplified dedifferentiated liposarcoma, in which an NTRK fusion was identified in tumors already harboring the defining genetic aberrations of those entities. Whether these sarcoma types are predisposed to acquiring NTRK fusions or whether their coexistence in this cohort represents a chance finding remains unknown. Nevertheless, these observations illustrate that NTRK fusions may occur in tumors with well-established canonical oncogenic drivers.

Taken together, these observations suggest that adult sarcomas harboring NTRK fusions may arise in at least two biological contexts. In one group, the NTRK fusion may represent the primary oncogenic driver, with additional genomic alterations contributing to tumor progression. In another group, particularly in tumors corresponding to established sarcoma entities, the NTRK fusion may function as a cooperating rather than initiating oncogenic event, although functional studies are required to establish oncogenic hierarchy. Importantly, genomic complexity should not be equated with loss of oncogenic dependence. Although secondary genomic alterations may accumulate, they do not necessarily preclude continued dependence on NTRK signaling or diminish the biological or therapeutic relevance of the fusion.

Clinical trial and real-world data indicate that responses to TRK inhibition can occur across diverse NTRK fusion isoforms in solid tumors [21,33,34,35]. In our retrospective series, however, clinical benefit was variable despite transcriptional activation of the fusion in all evaluable tumors. Some patients had prolonged treatment courses suggestive of durable benefit, whereas others had only brief exposure, including a patient with dedifferentiated liposarcoma treated sequentially with larotrectinib and entrectinib. We could not systematically assess intratumoral heterogeneity in NTRK fusion status (by FISH) and TRK protein expression (by immunohistochemistry) because additional tissue was unavailable, and future spatial assays will be needed to define how fusion-expression patterns relate to oncogenic dependence and treatment response. Nonetheless, these findings support the interpretation that NTRK fusion status alone does not invariably predict oncogenic dependence or uniform susceptibility to TRK inhibition in adult sarcomas and are consistent with emerging pan-cancer analyses suggesting that the relevance of NTRK fusions may be context dependent rather than uniform across fusion-positive tumors [36].

This study has several limitations. As a retrospective analysis derived from a referral-based molecular profiling database, the cohort is subject to selection bias and is likely enriched for clinically aggressive or diagnostically challenging tumors. The predominance of high-grade and advanced-stage disease may not represent the full clinical spectrum in unselected populations. The modest cohort size limits statistical power and precludes definitive correlations between genomic features and clinical response. Immunophenotypic profiles, including TRK immunohistochemistry, could not be systematically assessed; however, fusion-associated overexpression was demonstrated at the transcript level. Treatment response was inferred from real-world time on treatment, which serves as a pragmatic surrogate for treatment exposure and potential clinical benefit but does not fully capture radiographic response or standardized clinical response measures. In addition, reasons for TRK inhibitor discontinuation (e.g., disease progression, toxicity, financial or access constraints) could not be determined from claims data. Accordingly, time on treatment should not be interpreted as a validated surrogate for progression-free survival or objective response, and our observations regarding TRK inhibitor-associated outcomes in this cohort should be interpreted cautiously and not as definitive estimates of treatment efficacy.

## 5. Conclusions

Our study suggests that adult sarcomas harboring NTRK fusions are morphologically heterogeneous and frequently high grade, with diverse fusion partners and variable secondary genomic alterations. Although all evaluable tumors demonstrated transcriptional activation of the NTRK fusion and increased MAPK pathway activity, clinical benefit from TRK inhibition was heterogeneous, supporting a model in which NTRK fusions function in distinct biological contexts, serving as the dominant oncogenic driver in some tumors while acting as a cooperating alteration in others. NTRK fusion status alone may therefore not fully capture oncogenic dependence and should be interpreted within the broader clinicopathologic and genomic context to refine therapeutic decision-making in adult NTRK-rearranged sarcomas.

## Figures and Tables

**Table 1:** Clinical and demographic characteristics of adult sarcomas harboring NTRK fusions.

	Overall	NTRK1-Rearranged	NTRK3-Rearranged	Statistic	*p*-Value
**Age**					
Median age (range)	43.0 (21–77)	43.0 (24–68)	59.0 (21–77)	Mann–Whitney U	0.401
**Sex**					
Female	63.2% (12/19)	66.7% (6/9)	55.6% (5/9)	Fisher exact	>0.99
Male	36.8% (7/19)	33.3% (3/9)	44.4% (4/9)		
**Race**					
White	36.8% (7/19)	44.4% (4/9)	33.3% (3/9)	Fisher exact	>0.99
Black or African American	15.8% (3/19)	11.1% (1/9)	11.1% (1/9)		
Unknown/Other	47.4% (9/19)	44.4% (4/9)	55.6% (5/9)		
**Primary Site**					
Extremity	36.8% (7/19)	33.3% (3/9)	33.3% (3/9)	Fisher exact	0.615
Cervix	26.3% (5/19)	22.2% (2/9)	33.3% (3/9)		
Retroperitoneum/Abdomen	10.5% (2/19)	0.0% (0/9)	22.2% (2/9)		
Lung/Pleura	10.5% (2/19)	11.1% (1/9)	11.1% (1/9)		
Breast	10.5% (2/19)	22.2% (2/9)	0.0% (0/9)		
Head & Neck	5.3% (1/19)	11.1% (1/9)	0.0% (0/9)		
**Histologic Category**					
Spindle cell sarcoma	52.6% (10/19)	66.7% (6/9)	44.4% (4/9)	Fisher exact	0.876
Pleomorphic sarcoma	21.1% (4/19)	11.1% (1/9)	22.2% (2/9)		
Malignant peripheral nerve sheath tumor	10.5% (2/19)	11.1% (1/9)	11.1% (1/9)		
Dedifferentiated liposarcoma	10.5% (2/19)	11.1% (1/9)	11.1% (1/9)		
Sarcoma with epithelioid morphology	5.3% (1/19)	0.0% (0/9)	11.1% (1/9)		
**Histologic Grade**					
Low	10.5% (2/19)	0.0% (0/9)	22.2% (2/9)	Fisher exact	0.519
Intermediate	21.1% (4/19)	22.2% (2/9)	22.2% (2/9)		
High	68.4% (13/19)	77.8% (7/9)	55.6% (5/9)		
**Disease Stage**					
Localized	21.1% (4/19)	0.0% (0/9)	44.4% (4/9)	Fisher exact	0.022
Locally Advanced (includes local recurrences)	15.8% (3/19)	33.3% (3/9)	0.0% (0/9)		
Metastatic (Stage IV)	63.2% (12/19)	66.7% (6/9)	55.6% (5/9)		

**Table 2:** NTRK fusion partners and rearrangement characteristics in adult sarcomas.

Subgroup	NTRK Location	Fusion	Partner Location	Topology	Count
NTRK1-rearranged	1q23.1	*TPM3::NTRK1*	1q21.3	Inversion	5
		*ATF7-NPFF::NTRK1*	12q13.13	Translocation	1
		*MEF2D::NTRK1*	1q22	Inversion	1
		*TPR::NTRK1*	1q31.1	Inversion	1
		*NAV1::NTRK1*	1q32.1	Duplication	1
NTRK2-rearranged	9q21.33	*STRN::NTRK2*	2p22.2	Translocation	1
NTRK3-rearranged	15q25.3	*TFG::NTRK3*	3q12.2	Translocation	2
		*EML4::NTRK3*	2p21	Translocation	2
		*AKAP13::NTRK3*	15q25.3	Inversion	1
		*ARNT2::NTRK3*	15q25.1	Inversion	1
		*ETV6::NTRK3*	12p13.2	Translocation	1
		*HDAC4::NTRK3*	2q37.3	Translocation	1
		*SNX25::NTRK3*	4q35.1	Translocation	1

## Data Availability

The datasets used and/or analyzed during the current study are available from the corresponding author on reasonable request. The deidentified sequencing data are owned by Caris Life Sciences and cannot be publicly shared due to the data usage agreement signed between the investigator and Caris. Qualified researchers can apply for access to these summarized data by contacting the corresponding author and signing a data usage agreement with Caris.

## Supplementary Materials

The following supporting information can be downloaded at: https://www.mdpi.com/article/10.3390/cancers18152528/s1, Figure S1: Tumor mutational burden and MAPK pathway activity in adult NTRK-rearranged sarcomas; Table S1: Clinical and diagnostic characteristics of adult sarcomas harboring NTRK fusions; Table S2: Detailed morphologic characteristics of adult sarcomas harboring NTRK fusions.
