# Supplementary material for: Adult Sarcomas with NTRK Fusions: Clinicopathologic and Genomic Heterogeneity
Source: Cancers (Basel). 2026 Aug 6;18(15):2528. doi: 10.3390/cancers18152528 (PMC13465312; doi:10.3390/cancers18152528)
Supplement: Supplementary file 1 [file cancers-18-02528-s001.zip › cancers-4447836-supplementary.pdf]

cancers

MDPI

SUPPLEMENTARY MATERIAL

1. Molecular Profiling and Genomic Analysis

1.1. DNA and RNA Sequencing

Because this study utilized samples spanning several years, the methods (including materials and software) used for DNA and RNA profiling evolved over time. Initially, DNA was profiled using a 592-gene panel prior to adoption of whole exome sequencing (WES) in 2020, with targeted reporting of 720 clinically relevant genes. Whole transcriptome sequencing (WTS) was performed separately. In 2023, this approach was replaced by MI Tumor Hybrid, which analyzes RNA and DNA from the same total nucleic acid extraction. Nucleic acid was extracted using appropriate FFPE kits for RNA, DNA, or total nucleic acid. DNA sequencing was performed using a 592-gene panel on a NextSeq 500 System (RRID:SCR_014983) or by WES with enrichment of 720 clinically relevant genes on a NovaSeq 6000 System (RRID:SCR_016387) (Illumina, Inc., San Diego, CA). WTS was performed on a Novaseq 6000 System to an average of 60M reads. Raw WTS data was demultiplexed by Illumina Dragen BioIT accelerator, trimmed, counted, PCR-duplicates removed, and aligned to human reference genome (hg19 or hg38 depending on platform and time of testing) by STAR aligner (RRID:SCR_004463) [1]. For transcription counting, transcripts per million (TPM) molecules were generated using the Salmon expression pipeline (RRID:SCR_017036) [2]. For MI Tumor Seek Hybrid, RNA was labeled during first-strand cDNA synthesis by adapter sequences on the 5′ end of the cDNA primers, and next-generation sequencing was performed (720 clinically relevant genes were sequenced at high-depth). Sequencing data were extracted into split FASTQ files (RNA and DNA) for processing. DNA variants detected were mapped to reference genome hg38, and well-established bioinformatics tools such as Burrows-Wheeler Aligner (BWA 0.7.17) (RRID:SCR_010910), SamTools (RRID:SCR_002105), Pindel (RRID:SCR_000560), and snpEff (RRID:SCR_005191) were incorporated to perform variant calling and annotation. Filtering was performed to remove benign, low quality, and low depth variants or variants determined to be unreliable in several analyses like strand bias and repeat analysis. Genetic variants identified were interpreted by board-certified molecular geneticists and categorized as 'pathogenic', 'likely pathogenic', 'variant of unknown significance', 'likely benign', or 'benign', according to the American College of Medical Genetics and Genomics (ACMG) standards. Pathogenic and likely-pathogenic variants are counted as "reportable".

1.2. Fusion Detection

Clinically relevant fusions and/or splice variants were detected from WTS data using STAR-Fusion. A fusion was called "detected" if the number of junction reads + (3X number of spanning reads) ≥3. Non-clinical fusions and/or splice variants were called "detected" if the number of junction reads + (3X number of spanning reads) ≥7. Any results not meeting the criteria above were called "not detected".

1.3. Copy Number Amplifications

Copy number variations were determined using CNVkit (RRID:SCR_021917). Amplification status was determined for 325 clinically relevant genes. A gene was determined to be amplified if segment-level copies were ≥ 6 with the 95% prediction interval >3.5. Updated thresholds for *MDM2* and *MET* were applied as of Feb 2025, as follows: for *MDM2* and *MET*, segmented copies ≥6 and unsegmented copies ≥4.

1.4. Loss of Heterozygosity (LOH)

The 22 autosomal chromosomes were split into 552 segments (2-6 Mb in size) and the LOH of single nucleotide polymorphisms (SNPs) within each segment was calculated.

Caris WES data consist of approximately 250K SNPs spread across the genome (17 SNPs/Mb), with 200K from exonic regions and 50K from intronic regions (Agilent Technologies, Santa Clara, CA, USA). The MI Tumor Seek Hybrid assay uses a KAPA HyperChoice MAX 5Mb Baits SNP panel (Roche, Indianapolis, IN, USA). SNP distances of each region were compared to control distances of a non- LOH reference (NA12878; RRID:CVCL_7526) via Student's t-test. A region was called positive if the average distance was larger than 0.15 Mb and corrected p-value was less than 0.02. Segments excluded from the calculation of genomic LOH include those spanning ≥90% of a whole chromosome or chromosome arm and segments which are not covered by the SNP backbone and the WES panel. The final call of genomic LOH is based on the percentage of all 552 segments with observed LOH (High ≥16%, Low <16%). For MI Tumor Seek Hybrid only, an indeterminate result is reported if fewer than 3,000 SNPs can be read or sample depth <200x.

1.5. Tumor Mutational Burden (TMB)

TMB was measured by counting mutations found per tumor in the coding regions of genes analyzed (1.4 Mb for 592, 1.5 Mb for WES, and 25 Mb for 25 Mb for the Hybrid assay). For the 592-gene panel NGS assay, missense mutations were counted, and for WES/Hybrid, missense, nonsense, in-frame INDEL, and frameshift variants were counted. Filtering was performed to remove low quality and low depth variants or variants determined to be unreliable or unassociated with TMB. Presumed germline variants found in databases such as dbSNP (RRID:SCR_002338) and Genome Aggregation Database (gnomAD) (RRID:SCR_014964) and found in at least 10% of internal training samples were also filtered. A cutoff point of ≥10 mutations per megabase (Mb) was used based on the KEYNOTE-158 pembrolizumab trial [3], which showed that patients with a TMB of ≥10 mt/Mb across several tumor types had higher response rates than patients with a TMB of <10 mt/Mb. Samples with low depth of coverage (<400x for 592 or WES and <100x for Hybrid) were considered indeterminate. Caris Life Sciences is a participant in the Friends of Cancer Research TMB Harmonization Project [4].

1.6. Survival Analysis

Real-world overall survival (OS) information was obtained from insurance claims data. OS was calculated from time of tissue collection as a surrogate for diagnosis until death or last clinical contact. Patients without contact/claims data for a period of at least 365 days were presumed deceased. Patients without a confirmed death and with documented clinical activity within 365 days prior to the most recent data update were censored in the analysis. Time-on-treatment (TOT) was calculated from start of treatment with larotrectinib or entrectinib to end of treatment. Kaplan-Meier estimates were calculated separately for each molecularly defined group (e.g., by NTRK fusion status or fusion type). Hazard ratios (HR) were determined by Cox Proportional Hazards model and p-values by log-rank test. Significance was determined as $p < 0.05$.

1.7. Statistical Analysis

Statistical analysis was performed in Python 3.14 using the Numpy, Pandas, and Scipy packages. Significance was assessed using the chi-square test, Fisher exact test, or Mann-Whitney U test.

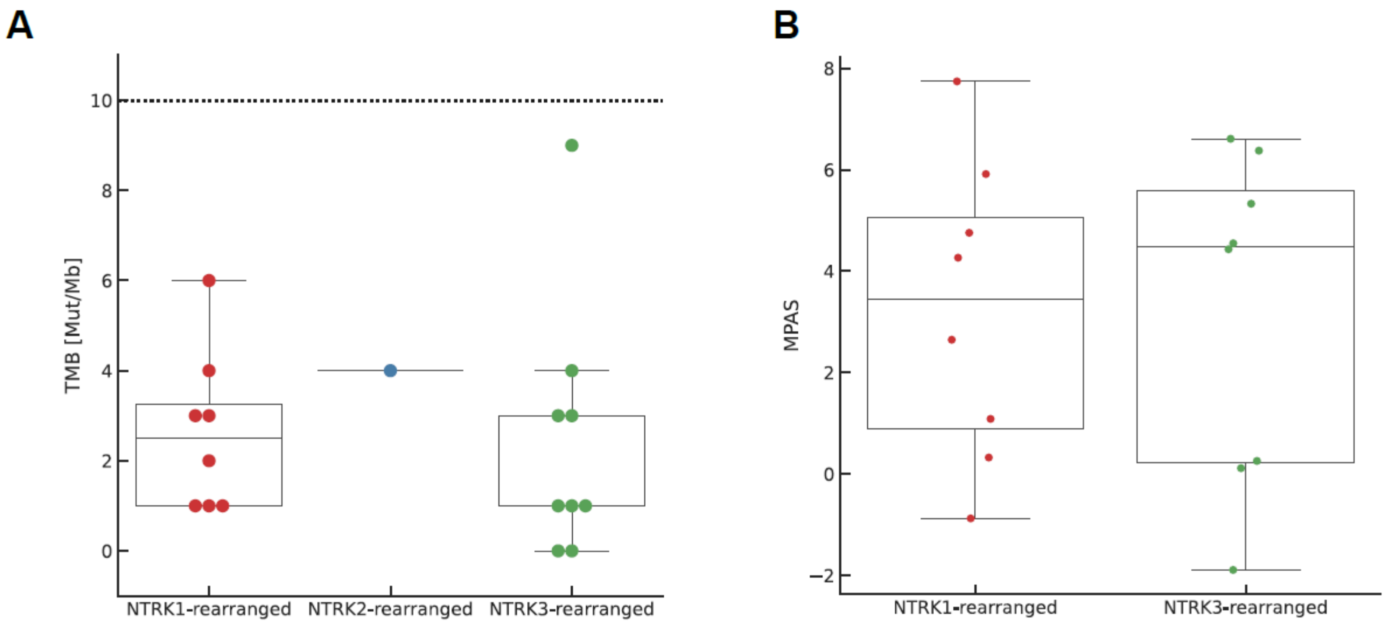

Figure S1. Tumor mutational burden and MAPK pathway activity in adult NTRK-rearranged sarcomas. A) Tumor mutational burden (TMB) across NTRK fusion subgroups. Each point represents an individual tumor. The dashed line denotes the threshold of 10 mutations/Mb used to define TMB-high. B) MAPK pathway activity score (MPAS) across NTRK fusion subgroups. Boxes represent interquartile ranges, center lines indicate medians, and whiskers denote the data range.

Table S1. Clinical and diagnostic characteristics of adult sarcomas harboring NTRK fusions

Case	Fusion	Age group	Sex	Specimen type	Histologic category
1	NTRK1	20-39 years	M	Metastatic	Spindle cell sarcoma, NOS
2	NTRK1	40-59 years	F	Metastatic	Spindle cell sarcoma, NOS
3	NTRK1	40-59 years	F	Local recurrent	Spindle cell sarcoma, NOS
4	NTRK1	≥60 years	F	Local recurrent	Spindle cell sarcoma, NOS
5	NTRK1	20-39 years	M	Primary	Malignant peripheral nerve sheath tumor
6	NTRK1	40-59 years	M	Metastatic	Pleomorphic sarcoma
7	NTRK1	20-39 years	F	Metastatic	Spindle cell sarcoma, NOS
8	NTRK1	20-39 years	F	Metastatic	Spindle cell sarcoma, NOS
9	NTRK1	40-59 years	F	Primary	Dedifferentiated liposarcoma
10	NTRK2	20-39 years	F	Metastatic	Pleomorphic sarcoma
11	NTRK3	≥60 years	M	Primary	Spindle cell sarcoma, NOS
12	NTRK3	20-39 years	F	Primary	Spindle cell sarcoma, NOS
13	NTRK3	20-39 years	F	Primary	Sarcoma with epithelioid morphology
14	NTRK3	20-39 years	F	Primary	Spindle cell sarcoma, NOS
15	NTRK3	20-39 years	M	Metastatic	Malignant peripheral nerve sheath tumor
16	NTRK3	Not specified	M	Local recurrent	Dedifferentiated liposarcoma
17	NTRK3	≥60 years	M	Metastatic	Pleomorphic sarcoma
18	NTRK3	40-59 years	F	Primary	Pleomorphic sarcoma
19	NTRK3	≥60 years	F	Primary	Spindle cell sarcoma, NOS (with myxoid features)

Table S2. Detailed morphologic characteristics of adult sarcomas harboring NTRK fusions

Case	Grade	Predominant cytomorphology	Cytologic atypia	Diffuse nuclear pleomorphism	Mitotic activity (semiquantitative)	Predominant growth pattern and stromal features	Tumor necrosis
1	3	Spindle	Severe	No	Brisk	Long and short fascicular	Absent
2	3	Spindle	Moderate	No	Brisk	Long fascicular	Present, geographic (<50%)
3	2	Spindle	Moderate	No	Brisk	Short fascicular in a hyalinized and focally myxoid stroma	Absent
4	2	Spindle and ovoid	Moderate	No	Intermediate	Short fascicular with mixed inflammatory cells	Absent
5	3	Spindle	Severe	No	Brisk	Long fascicular (adjacent to neurofibroma); vascular invagination	Present, focal (<50%)
6	3	Spindle and pleomorphic	Severe	Yes, pronounced	Brisk	Haphazard	Present (<50%)
7	3	Spindle	Severe	No	Brisk	Long and short fascicular	Present, focal (<50%)
8	3	Spindle and ovoid	Severe	No	Brisk	Short fascicular and haphazard	Present (<50%)
9	3	Spindle	Severe	Present, moderate	Brisk	Short fascicular (adjacent well-differentiated liposarcoma)	Present (>50%)
10	3	Spindle and pleomorphic	Severe	Yes, pronounced	Brisk	Haphazard in myxoid stroma	Present (<50%)
11	3	Spindle	Moderate	No	Brisk	Short fascicular with packeted arrangement	Absent
12	1	Spindle	Mild	No	Low	Swirling, storiform, and infiltrative	Absent
13	2	Epithelioid and ovoid	Moderate	No	Brisk	Swirling and packeted	Absent
14	1	Spindle	Moderate	No	Intermediate	Swirling, storiform, and infiltrative	Absent
15	3	Spindle	Severe	No	Brisk	Fascicular; vascular invagination; variably myxoid stroma	Present (<50%)
16	3	Spindle and pleomorphic	Severe	Yes, pronounced	Intermediate	Haphazard (adjacent well-differentiated liposarcoma)	Absent
17	3	Spindle and pleomorphic	Severe	Yes, pronounced	Brisk	Haphazard	Present (<50%)
18	3	Spindle and pleomorphic	Severe	Yes, pronounced	Brisk	Haphazard	Present (<50%)
19	2	Spindle and ovoid	Moderate	No	Intermediate	Short fascicular in myxoid stroma	Absent

References

1. Dobin, A.; Davis, C.A.; Schlesinger, F.; Drenkow, J.; Zaleski, C.; Jha, S.; Batut, P.; Chaisson, M.; Gingeras, T.R. STAR: Ultrafast universal RNA-seq aligner. *Bioinformatics* **2012**, *29*, 15–21, doi:10.1093/bioinformatics/bts635.
2. Patro, R.; Duggal, G.; Love, M.I.; Irizarry, R.A.; Kingsford, C. Salmon provides fast and bias-aware quantification of transcript expression. *Nat. Methods* **2017**, *14*, 417–419, doi:10.1038/nmeth.4197.
3. Marabelle, A.; Fakih, M.; Lopez, J.; Shah, M.; Shapira-Frommer, R.; Nakagawa, K.; Chung, H.C.; Kindler, H.L.; Lopez-Martin, J.A.; Miller, W.H., Jr.; et al. Association of tumour mutational burden with outcomes in patients with advanced solid tumours treated with pembrolizumab: prospective biomarker analysis of the multicohort, open-label, phase 2 KEYNOTE-158 study. *Lancet Oncol.* **2020**, *21*, 1353–1365, https://doi.org/10.1016/s1470-2045(20)30445-9.
4. Merino, D.M.; McShane, L.M.; Fabrizio, D.; Funari, V.; Chen, S.J.; White, J.R.; Wenz, P.; Baden, J.; Barrett, J.C.; Chaudhary, R.; et al.; TMB Harmonization Consortium. Establishing guidelines to harmonize tumor mutational burden (TMB): In silico assessment of variation in TMB quantification across diagnostic platforms: Phase I of the Friends of Cancer Research TMB Harmonization Project. *J. Immunother. Cancer* **2020**, *8*, e000147. doi:10.1136/jitc-2019-000147.

Disclaimer/Publisher's Note: The statements, opinions and data contained in all publications are solely those of the individual author(s) and contributor(s) and not of MDPI and/or the editor(s). MDPI and/or the editor(s) disclaim responsibility for any injury to people or property resulting from any ideas, methods, instructions or products referred to in the content.
